# Inter-rater reliability of the Silverman and Andersen index-a measure of respiratory distress in preterm infants

**DOI:** 10.1371/journal.pone.0286655

**Published:** 2023-06-30

**Authors:** Hilde Brenne, Turid Follestad, Håkon Bergseng, Beate Horsberg Eriksen, Karin Søraunet, Kristine Hermansen Grunewaldt

**Affiliations:** 1 Department of Pediatrics, St. Olavs Hospital Trondheim University Hospital, Trondheim, Norway; 2 Department of Clinical and Molecular Medicine, Norwegian University of Science and Technology, Trondheim, Norway; 3 Clinical Research Unit, Norwegian University of Science and Technology, Trondheim, Norway; 4 Department of Pediatrics, Ålesund Hospital, Ålesund, Norway; 5 Department of Pediatrics, Levanger Hospital, Levanger, Norway; Stanford University School of Medicine, UNITED STATES

## Abstract

**Background:**

There are various methods of respiratory support available to optimize respiratory function in preterm infants. Respiratory scoring tools might provide information on which method to choose and the level and duration of support needed. Before implementing a respiratory scoring tool in our clinical practice, we aimed to test the inter- and intra-rater reliability of the Silverman and Andersen index (SA index) among neonatologists and nurses when applied to preterm infants on respiratory support. We also examined the association between the SA index and the electrical activity of the diaphragm (Edi signals).

**Methods:**

This was a multicenter study including three newborn intensive care units in Norway. Four neonatologists and 10 nurses applied the SA index when assessing 80 videos of 44 preterm infants on High Flow Nasal Cannula, Continuous Positive Airway Pressure and Neurally Adjusted Ventilatory Assist. The inter- and intra-rater reliability for the sum scores were measured by the intra-class correlation coefficient (ICC), and Kendall’s W was used to assess the degree of agreement for each item. We quantified the association between the Edi signals and the SA index scores by the Spearman’s correlation coefficient.

**Results:**

We found poor inter-rater reliability with an ICC for absolute agreement of 0.34 (95% CI: 0.20 to 0.53). There was fair agreement measuring each item separately for upper chest movements (Kendall’s W 0.30), and moderate for lower chest movements (0.43) and xiphoid retractions (0.44). Expiratory grunting showed substantial agreement (0.67). The intra-rater reliability was good (ICC for absolute agreement 0.77; 95% CI: 0.68 to 0.84). We found a moderate positive correlation (r = 0.468, p = 0.028) between the maximum inspiratory diaphragm activity (Edi peak) and the mean inspiratory SA index scores.

**Conclusion:**

Our study showed poor inter-rater and good intra-rater reliability of the SA index when nurses and neonatologists assessed videos of preterm infants on various types of respiratory support. Edi peak and SA index had a moderate positive correlation. Formal training might be essential to improve the inter-rater reliability.

**Trial registration:**

Registered 26^th^ June 2017, ClinicalTrials.gov Identifier: NCT03199898.

## Introduction

Healthcare providers in newborn intensive care units (NICU`s) are faced with the challenge of choosing the optimal respiratory support in preterm infants [1, 2]. Respiratory distress is a common complication to preterm birth caused by several factors related to immaturity of the respiratory centers, the upper and lower airways as well as muscle strength [3]. Preterm infants might need days and even weeks on respiratory support [4]. Various modes of respiratory support systems make it possible to switch methods to prevent over- or under ventilation [2, 3]. Optimizing the respiratory care aims to reduce the risk of bronchopulmonal dysplasia, long-term developmental problems and death [1, 5].

**Fig 1:**
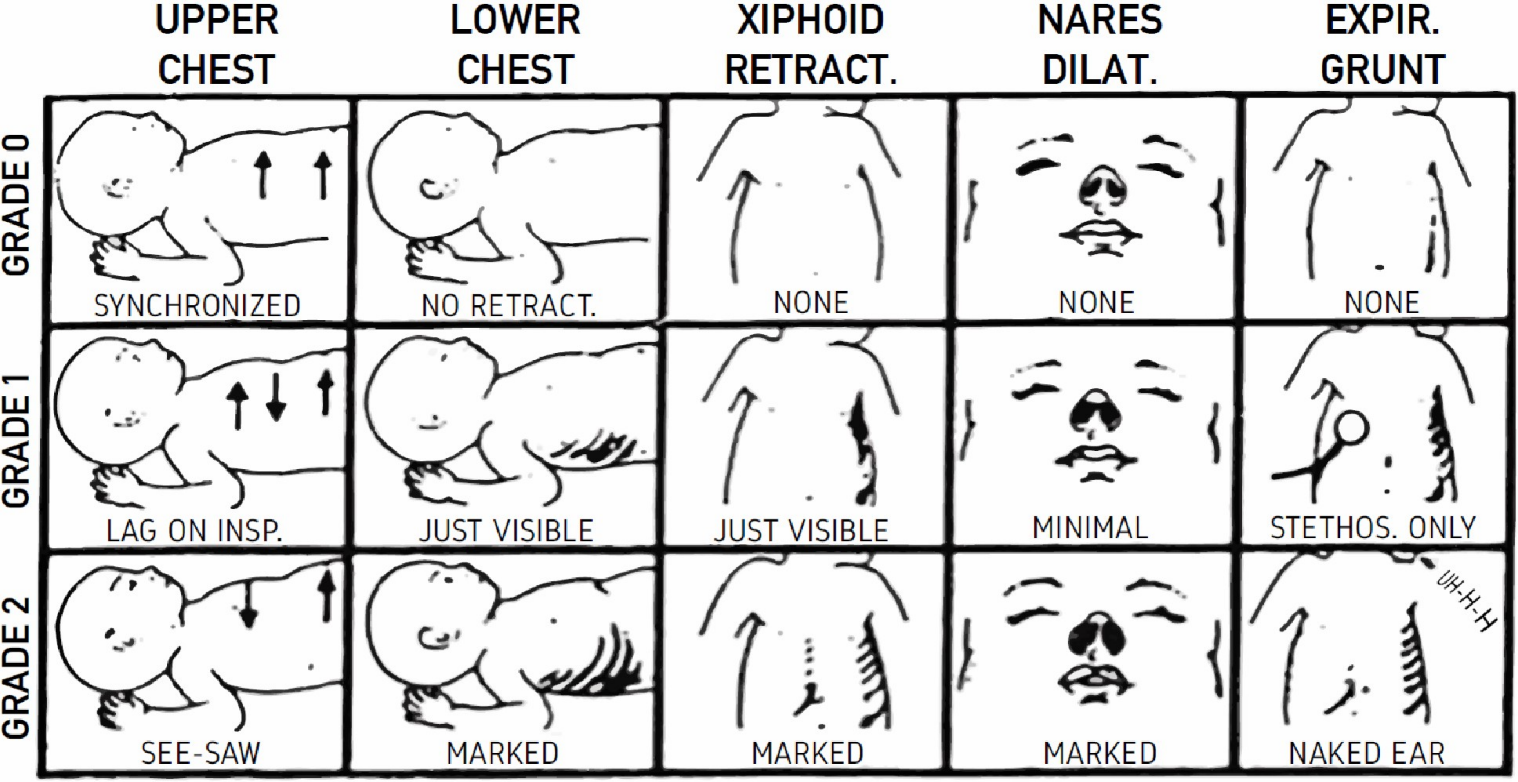
The Silverman and Andersen index. Reproduced with permission from Public Domain Courtesy of Hathi Trust https://catalog.hathitrust.org/Record/002069837, Dunham EC, Silverman WA. Dunham’s Premature infants, 1961.

Assessment of classical symptoms of respiratory distress such as chest and abdominal retractions, nasal flaring, grunting and tachypnea might be easily recognizable [6, 7], though associated symptoms like bradypnea, apnea, paradoxical respiratory patterns, cyanosis, and generally increased work of breathing, might be more difficult to grade accurately. Observational scoring tools can provide important information to help decide when to start weaning from respiratory support, when to increase support and how to identify those infants at risk of impending respiratory failure [8]. The Silverman and Andersen index (SA index) is a graded description of five items of the symptoms of respiratory distress in preterm infants that is easy to perform [9, 10] “Fig 1”.

In a former study evaluating the use of SA index in the clinical routine, the authors found only slight inter-rater agreement among the raters when assessing preterm infants breathing on various types of non-invasive ventilation (NIV) [11]. The authors speculated that the SA index was inaccurate due to the difficulty in observing nasal flaring when infants wear an interface with masks or prongs [11]. Setty et al. removed the indices of nares dilation and expiratory grunt and found that this simplified versions of the SA index had a predictive value in line with the full SA index [12]. Performing the SA index is considered relatively simple, though training might still be important to execute the assessment accurately. Cavallin et al. showed that training improved agreement and made it easier to identify not only severe, but also mild to moderate respiratory distress [13]. A review of scoring tools at different ages showed a low reliability in 60% of the investigated tools [8]. In regard to the SA index, studies investigating reliability are limited. Prior to implementing a respiratory scoring tool in our clinical practice, we aimed to assess the inter- and intra-rater reliability of the SA index among neonatologists and nurses when applied to preterm infants on various modes of respiratory support. We also aimed to measure the association between electrical activity in the diaphragm (Edi signals) and the SA index.

## Methods

**Fig 2:**
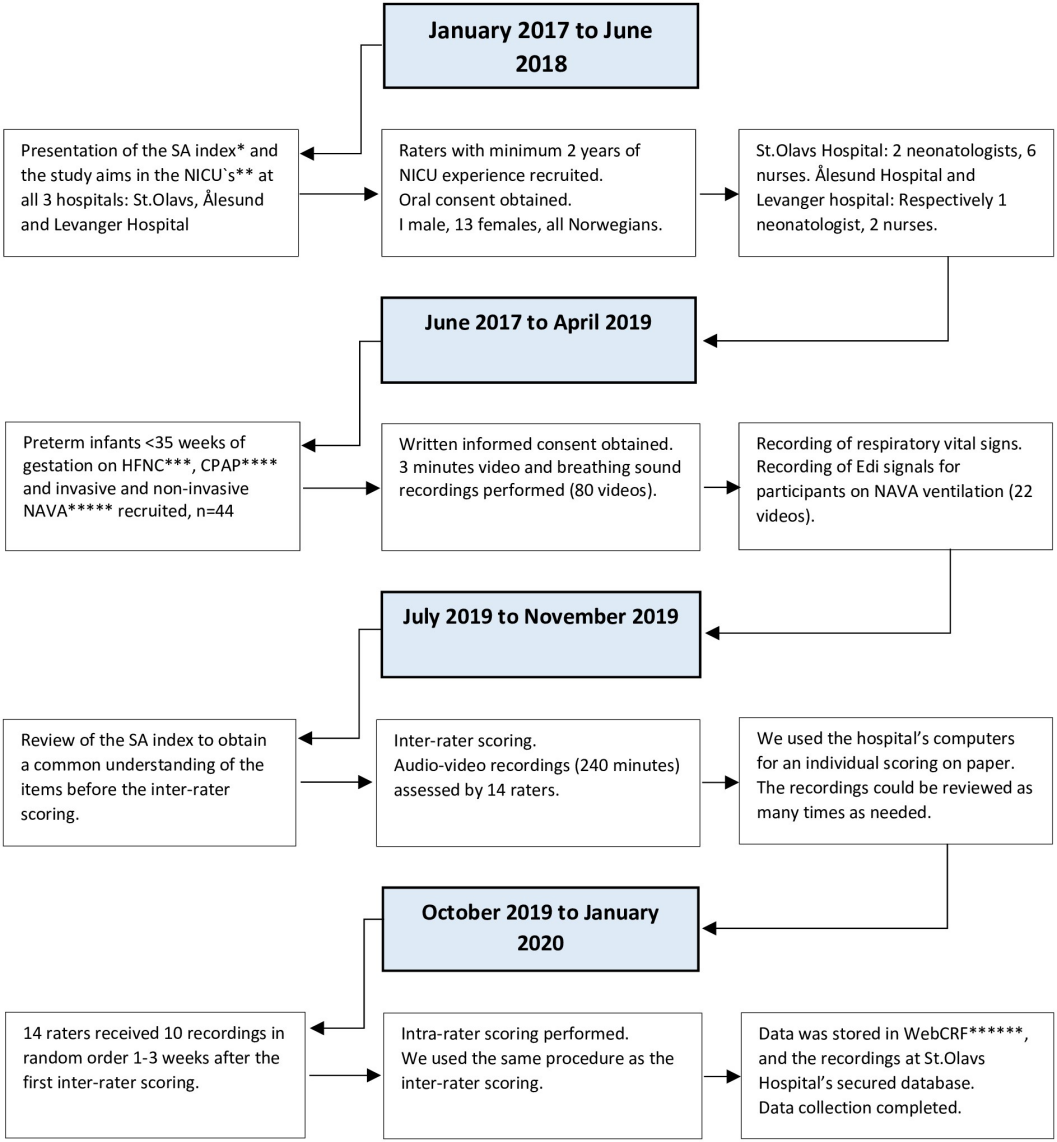
The process over the study period. *Silverman and Andersen index. **Newborn Intensive Care Unit. ***High Flow Nasal Cannula. ****Continuous Positive Airway Pressure. *****Neurally Adjusted Ventilatory Assist. ******Internet Based Data Retrieval.

This study was performed at St. Olavs hospital Trondheim University Hospital, Ålesund Hospital and Levanger Hospital. Preterm infants born at St. Olavs hospital with gestational age (GA) below 35 weeks breathing on High Flow Nasal Cannula (HF), Continuous Positive Airway Pressure (CPAP), mechanical ventilation or invasive or non-invasive Neurally Adjusted Ventilator Assist (NAVA/NIV NAVA), were included from June 2017 to April 2019 “Fig 2”. A total of 80 videos were recorded within the first weeks of life. GA, postmenstrual age, weight at birth and at video recordings were collected from the patients’ journal. Infants with hemodynamic instability, pneumothorax or in need of muscle relaxation were excluded from this study.

By a formal request to the head of the NICUs, 10 nurses, one neonatologist and three senior consultant neonatologists with more than two years of experience in the NICU were recruited as raters. They gave verbal consent for participation, and we planned the inter-rater scoring during work time with no compensation. None of the raters had previously used the SA index in their clinical practice. Despite this, the items in the SA index represent the classical symptoms of respiratory distress [6], and is one of the main observations in the NICU well known to the nurses and neonatologists. The raters were blinded to the medical history of the infants. In the preparation for the study, each NICU was introduced to the SA index by a study coordinator (HB), going through the items in the scoring tool and the purpose of the study at departmental meetings. In addition, each rater had an interactive review of the different chest movements and expiratory sounds before the inter-rater scoring for a common understanding of how to use the scoring tool. The process of the study period is shown in “Fig 2”. The Regional Committee for Medical Research Ethics in Central Norway approved the study (registration number 2017588), and written, informed parental consent was obtained prior to enrollment.

### Video and breathing sound recordings

The study coordinator (HB) used a Samsung NX 3000, 20 mega pixels camera (Samsung Electronics, Surrey, United Kingdom) for video recording. The incubator mattress was elevated at a 30% angle, and a three-minute recording was performed at minimum one hour after feeding showing the preterm infant’s body, chest and abdomen, face, nose and the auscultation with a Littman Electronic Stethoscope 3200 (3M Health Care D-41453 Neuss Germany). Diaphragm mode was set at 20–2000 hertz when recording the breathing sounds, and the amplification level to III. Each video was de-identified and stored with the VideoLAN Movie Creator for Windows version 3.0.10 (VideoLAN, 70013 Paris, France) on St. Olavs hospital`s secured database.

### Inter- and intra-rater scoring

There were 240 minutes of video-recordings, and the raters could see the videos and listen to the breathing sounds as many times as needed using a memory stick on the hospital computers. An individual assessment was performed on paper that was stored and secured in the hospital together with the memory stick. The raters used Koss Porta Pro headphones (Tura Scandinavia AB, Kungsbacka, Sweden) to assess the breathing sounds. Ten videos and breathing sounds were selected for intra-rater scoring, and this assessment was performed one to 3 weeks after the inter-rater scoring.

### Measures

The SA index reflects the severity of respiratory distress consisting of 5 items (upper and lower chest movements, xiphoid retractions, nares dilation and expiratory grunt) allocated from zero to two points for each item. Zero points represents no symptoms, one point represents moderate respiratory effort, and two points represents maximum respiratory effort. The sum score is therefore in the range from zero to 10 [10].

Using NAVA, micro sensors on an advanced feeding tube will capture the Edi signals representing maximum inspiratory diaphragm activity (Edi peak) and the tone in the diaphragm between the breaths (Edi min) [14, 15]. These signals trigger the ventilator to give synchronized respiratory support [14]. The Edi signals were transferred in 30-second intervals simultaneously with the video recordings from the Servo ventilator (Maquet Critical Care AB, Solna, Sweden) together with the respiratory rate, heart rate and saturation from the IntelliVue MP 50 (Philips Healthcare, Andover, MA, USA) to the Critical Care Manager 8.0 computer-based information system (Picis, Wakefield, MA, USA).

### Statistical method and sample size

Continuous variables (e.g. respiratory rate of the infants) are presented as mean and standard deviation (SD) or median and range as appropriate. Categorical variables (e.g. gender of the infants) are presented as frequencies and percentages. Considering the data as continuous, the inter-rater reliability for the sum scores of the SA index was quantified by the intra-class correlation (ICC) using a two-way random effects model [16]. The ICC for absolute agreement as well as for consistency were calculated based on this model, and presented with 95% confidence intervals (CIs) [16]. We also analyzed the sum scores separately for the nurses and the neonatologists, and the preterm infants separately on CPAP, HFNC and NIV NAVA.

We calculated the ICC for absolute agreement and for consistency for the intra-rater reliability in a similar way based on a two-way mixed-effects model. ICC < 0.5 is interpreted as poor, 0.50–0.75 as moderate, 0.75–0.90 as good, and ICC > 0.90 as excellent reliability [17, 18]. The degree of agreement in each individual item of the SA index was measured by Kendall’s Coefficient of Concordance (W) and the strength of agreement is interpreted as < 0 poor, 0.00–0.20 slight, 0.21–0.40 fair, 0.41–0.60 moderate, 0.61–0.80 substantial, and 0.81–1.00 almost perfect agreement [19].

In 22 of the video recordings, the infants were ventilated on NAVA. We used the Spearman correlation as a measure of association between Edi peak and the mean of the inspiratory scores of the SA index, and between Edi min and the expiratory grunt. The statistical analyses were performed using the SPSS statistical software program version 25.0 (IBM Corp, New York, NY, USA).

Due to missing data “Table 1”, the ICC for inter-rater reliability was calculated in several ways: by complete-case analysis (i.e. using sum scores only where all items are observed), by imputing the mean of observed items for missing values for one or two items (nares dilation and/or expiratory grunting) and by removing the items of nares dilation and expiratory grunt. For the remaining ICC calculations, including those for intra-rater reliability, complete case analysis was used, but without the item of nares dilation analyzing infants with CPAP and NAVA separately.

**Table 1:** Number of observed and missing items.

Number of items (n = 1120)	Observed items (n)	Missing items (n)
Upper chest	1115	5
Lower chest	1114	6
Xiphoid retraction	1115	5
Nares dilated	811	309
Expiratory grunt	1085	35

Data expressed as frequencies, number

The assessments were based on 80 video recordings. According to COnsensus-based Standards for the selection of Health Measurement INstruments (COSMIN) [20] and the COSMIN checklist with 4-point scale, the recommended sample size of 50–99 subjects would ensure a study of good quality. In our study with 80 videos of 44 infants, we consider the videos as the subjects. Thirty-six recordings were found to be sufficient to estimate an ICC of 0.75 with a 95% CI of length 0.2, that is, a 95% CI from 0.65 to 0.85, when using n = 14 raters, adjusting for a 10% drop-out [21, 22]. The determination of the final sample size was based on a pragmatic approach using the COSMIN guidelines and the sample size calculations.

## Results

In this study, 55 infants were eligible to participate. Three infants were off respiratory support and parents of eight infants did not give their consent to participate, hence 44 infants were included. The raters were 13 females and one male, all Norwegian citizens. The nurses had a median work experience of 14.5 (range 9–30) years, and the neonatologists 16 (range 4–23) years.

The characteristics of the preterm infants are presented in “Table 2”.

**Table 2:** Summary of infant characteristics, vital signs and type of respiratory support.

Characteristics		
Gestational age (week^days^), median (range)	28 (23^3^−34^3^)	
Birthweight (g), median (range)	1072 (500–3100)	
Gender (girs/boys), n	21/23	
	**Video 1**	**Video 2**
Postmenstrual age, median (range)	30^2^ (26^4^−35^5^)	31^1^ (27^1^−38^4^)
Weight at study (g), median (range)	1252 (770–2575)	1461 (915–2895)
Respiratory rate, mean (SD)	52 (11)	52 (12)
Heart rate, mean (SD)	156 (14)	164 (16)
SaO_2_*, mean (SD)	95 (4)	94 (4)
	**Type of respiratory support, n (%)**	
High Flow Nasal Cannula	29 (36)	
Continuous Positive Airway Pressure	27 (34)	
Biphasic Continuous Positive Airway Pressure	1 (1)	
Non-invasive Neurally Adjusted Ventilator Assist	15 (19)	
Invasive Neurally Adjusted Ventilatory Assist	7 (9)	
Conventional ventilation	1 (1)	

*Saturation of oxygen

The inter-rater reliability for the sum scores of the SA index among neonatologists and nurses was poor, both as measured by ICC for absolute agreement (0.34; 95% CI: 0.20 to 0.53) and for consistency (0.51; 95% CI: 0.37 to 0.68). The number of videos analyzed and those excluded are found in “Table 3”. The ICCs indicate poor reliability for all these additional inter-rater analyses “Table 3”.

**Table 3:** Different reliability measures of inter- and intra-rater scorings of the SA index.

	ICC Agreement (95% CI)	ICC Consistency (95% CI)	Number of videos analyzed, n (%)	Number of videos excluded, n (%)
**Inter-rater reliability, sum scores, among all raters**				
All items	0.34 (0.20 to 0.53)	0.51 (0.37 to 0.68)	25 (31)	55 (69)
Without the item nares dilation	0.34 (0.22 to 0.47)	0.52 (0.43 to 0.63)	63 (79)	17 (21)
Without the items nares dilation and expiratory grunting	0.32 (0.21 to 0.44)	0.49 (0.40 to 0.58)	75 (94)	5 (6)
Imputing the mean of observed items for missing values for one or two items	0.33 (0.22 to 0.46)	0.51 (0.43 to 0.61)	75 (94)	5 (6)
**Inter-rater reliability, sum scores**				
Among nurses, all items	0.37 (0.21 to 0.57)	0.57 (0.43 to 0.73)	26 (33)	54 (68)
Among neonatologists, all items	0.26 (0.07 to 0.46)	0.43 (0.27 to 0.59)	42 (53)	38 (48)
Preterm infants on CPAP, without item of nares dilation	0.34 (0.19 to 0.56)	0.55 (0.39 to 0.73)	20 (74)	7 (26)
Preterm infants on HFNC, all items	0.36 (0.20 to 0.57)	0.54 (0.39 to 0.73)	20 (69)	19 (31)
Preterm infants on NAVA, without item of nares dilation	0.38 (0.22 to 0.61)	0.56 (0.39 to 0.76)	17 (77)	5 (23)
**Intra-rater reliability, Sum scores, all items**				
Among all raters	0.77 (0.68 to 0.84)	0.77 (0.68 to 0.84)	100 (71)	40 (29)
Among nurses	0.75 (0.62 to 0.84)	0.76 (0.63 to 0.84)	69 (69)	31 (31)
**A**mong neonatologists	0.87 (0.75 to 0.93)	0.87 (0.75 to 0.93)	31 (78)	9 (23)

The Intra-class correlation (ICC) was calculated by using a two-way random effects model for the inter-rater reliability and a two-way mixed-effects model for the intra-rater reliability. An ICC < 0.5 is interpreted as poor, 0.50–0.75 as moderate, 0.75–0.90 as good, and ICC > 0.90 as excellent reliability [17, 18] “S1 Dataset”

Assessing the agreement among all raters of the five items separately, Kendall’s W indicated fair agreement for the upper chest movements (0.30), and moderate agreement for the lower chest movements (0.43) and xiphoid retractions (0.44) “Table 4”. Expiratory grunt showed substantial agreement (0.67). The item of nares dilation was not assessed due to the large amount of missing data.

**Table 4:** Inter-rater scoring of each item of the SA index.

Inter-rater scoring of each item of the SA index	Kendall`s Coefficient of Concordance (W)
Upper chest movements	0.30
Lower chest movements	0.43
Xiphoid retraction	0.44
Nares dilated	Not measured due missing data
Expiratory grunt	0.67

Kendall’s Coefficient of Concordance (W) and the strength of agreement is interpreted as < 0 poor, 0.00–0.20 slight, 0.21–0.40 fair, 0.41–0.60 moderate, 0.61–0.80 substantial, and 0.81–1.00 almost perfect [19]

The intra-rater reliability for the sum score was in the area of good agreement with an ICC of 0.77 (95% CI: 0.68 to 0.84). When analyzing the nurses and neonatologists separately, both groups showed good intra-rater agreement with an ICC of 0.87 (95% CI 0.75 to 0.93) for physicians and 0.75 (95% CI 0.62 to 0.84) for nurses. Spearman’s rho showed a moderate positive association (r = 0.468, p = 0.028) between Edi peak and the mean of the inspiratory scores. There was no evidence of an association between Edi min and the expiratory grunt (r = 0.073, p = 0.747).

## Discussion

The main finding of this study was the poor inter-rater agreement among all raters in the sum scores of the SA index. Analyzing each item of the SA index separately, we found moderate agreement for the lower chest movements and xiphoid retractions. The intra-rater reliability was good and there was a moderate positive correlation between the inspiratory items of the SA index and the Edi peak signals.

The poor inter-rater agreement indicates that the use of the SA index might not provide enough information to identify when to scale up and when to start weaning from respiratory support. Nussbaum et al. showed that low SA index scores did not necessarily lead to weaning from NIV, yet higher scores were predictive against weaning [11]. The authors highlighted that bradycardias, apneas and desaturations might have a higher impact on the weaning decision than low scores of respiratory distress [11]. It is possible that the SA index is more useful in an “either—or” situation regarding whether the preterm infants need respiratory support or not.

For some video recordings, we found a wide range of scores on the same video, assessed by the individual raters, from mild to moderate and even severe respiratory difficulty. This illustrates the complexity of such clinical assessments and the individual perception of the observer. We speculate that the SA index might be too imprecise or not comprehensive enough when applied to preterm infants on various types of respiratory support and might give only a rough estimate of the respiratory state.

Peng et al. showed that the inter-rater agreement prior and after training showed persistent poor inter-rater agreement in adults with acute respiratory distress syndrome [23]. This was found even though training material consisting of a “gold standard” of examples set by an experienced panel from different countries was present [23]. These results underline that the perception of the severity of respiratory distress is challenging and subjective, and differs even for the more experienced clinicians.

Studies have shown that training in the skills in the use of SA index might contribute to easier implementation of CPAP in low- and middle-resource settings [13, 24]. To investigate whether training in the use of SA index could improve agreement among the raters, was not the scope of our study. Hence, as long as there are no “gold standards” in what level of work of breathing is acceptable at different gestational ages, this is challenging. The raters in our study had an introduction to the scoring tool and a review of the items for a common understanding, but no formal training prior to the study. This might have influenced our results negatively.

Jensen et al. investigated the inter-observer reliability among physicians performing a respiratory physical examination in preterm infants [25]. They found a lack of agreement in findings of visual and acoustic evaluation of respiratory symptoms. However, the highest inter-rater reliability was shown for the subcostal retractions, suprasternal retractions and head bobbing. In our study, we found a moderate agreement in the items of lower chest movements and xiphoid retractions. We speculate that the respiratory work of the lower chest parts might be easier to observe and evaluate than the upper chest parts. The presence of the rib margins during inspiration are more defined partly due to the compliant chest-wall and often distended abdomen during CPAP treatment in preterm infants [26–28].

The appearance of respiratory distress in infants might depend on associated risk burden, behavioral state, maturation, growth parameters as well as the specific type of respiratory support [27, 29]. In addition, the interface, masks, prongs and the endotracheal tube attachment might cover the nose or parts of the face, and hence make the observations of the nares dilation difficult [11]. Nasal flaring, expiratory grunting and chest retractions are some of the most common principal signs of respiratory distress in newborns [6]. In our study, the respiratory rate, heart rate and saturation did not indicate that the preterm infants were having breathing difficulties during the 3 minutes of video recordings, though other symptoms of respiratory distress like chest retractions were still observed when assessing the videos. This might indicate that the assessment of respiratory muscle work and respiratory monitoring gives conflicting information when estimating the respiratory difficulty in preterm infants.

In our study, we found a moderate positive correlation between the mean inspiratory scores of the SA index and the Edi peak. This underlines the fact that when the inspiratory effort increases, so does the Edi peak showing that there is an association between the inspiratory muscle work of the chest and the inspiratory electromyography signals from the diaphragm. NAVA and Edi signals give valuable information about several aspects of respiratory drive and effort [30] and Stein et al. showed that the Edi signals were consistent throughout maturation over one to 10 weeks in stable preterm infants with a post menstrual age of 26 to 33 weeks [15]. Edi signals and respiratory scoring tools would therefore be interesting to investigate in future studies.

### Strengths and limitations

There are several limitations in our study. Even if we had an introduction period and reviewing the SA index for a common understanding, we believe a more structured training protocol including an evaluation of training fidelity [31] might have improved the inter-rater reliability. For the future this is an important topic to consider when planning reliability studies [31]. A strength was that the raters represented experienced nurses and neonatologists from three different hospitals, and with extensive knowledge in the field of ventilation of preterm infants.

The SA index is originally a scoring tool for in-person assessment [9]. In our study, several raters from different hospitals evaluated the same assessments of infants to measure inter-rater reliability. We consequently had to perform video assessments and not in-person assessments. We cannot consider in-person and video assessment as identical and due to additional technical challenges when recording the infants; this might have been an important limitation.

The proportion of missing data of the item of nares dilation during non-invasive respiratory support represents another limitation. In our study, 42 videos of infants on CPAP or NIV NAVA were recorded where the interface with prongs or masks made it difficult or even impossible to observe the nose of the infants. A further limitation is that when evaluating 80 videos consisting of 240 minutes of recordings, a great amount of concentration is required. It is possible that raters have experienced fatigue during the long scoring period [32]; a problem that was not addressed prior to the study. It was up to the raters to take breaks when necessary and a fatigue might have influenced our results negatively.

## Conclusion

Our study showed poor inter-rater and good intra-rater reliability of the SA index when nurses and neonatologists assessed videos of preterm infants on various types of respiratory support. Edi peak and the mean of the inspiratory scores of the SA index had a moderate positive correlation. For future reliability studies, a formal training protocol measuring the fidelity of training might improve the inter-rater reliability.

## Abbreviations

NICUs: Newborn intensive care units
SA index: Silverman and Andersen index
NAVA: neurally adjusted ventilatory assist
NIV NAVA: non-invasive neurally adjusted ventilator assist
CPAP: continuous positive airway pressure
Edi signals: electrical activity of the diaphragm
Edi min: tone in the diaphragm between the breaths
Edi peak: maximum inspiratory diaphragm activity
SD: standard deviation
CIs: confidence intervals
